# Modelling the mass adoption potential of food waste composting among rural Chinese farmers

**DOI:** 10.1016/j.heliyon.2023.e18998

**Published:** 2023-08-09

**Authors:** Abdullah Al Mamun, Qing Yang, Farzana Naznen, Norzalita Abd Aziz, Muhammad Mehedi Masud

**Affiliations:** a UKM - Graduate School of Business, Universiti Kebangsaan Malaysia, UKM, Bangi, 43600, Selangor Darul Ehsan, Malaysia; b UCSI Graduate Business School, UCSI University, Cheras, 56000, Kuala Lumpur, Malaysia; c Faculty of Business and Economics, University of Malaya, 50603, Kuala Lumpur, Malaysia

**Keywords:** Food waste composting, Theory of interpersonal behavior, Farmers, China

## Abstract

As a safe alternative to hazardous agrochemicals, food waste compost could prevent human health hazards and environmental degradation. Food waste composting has not garnered much popularity among farmers given their sole dependence on synthetic fertilizers for high yields and commercial returns. Hence, this study aimed to identify the factors influencing farmers’ adoption of food waste composting for regular use. Empirical data were collected from 399 farmers residing in different second-tier cities in China through face-to-face interviews using a structured questionnaire. The partial least squares-structural equation modelling was used to statistically examine the models and construct correlations. Based on the study outcomes, the perceived usefulness of food waste compost, awareness of the consequences, social influence, anticipated guilt, and attitude towards food waste composting substantially impacted food waste composting intention. Intriguingly, the perceived value of sustainability and ascription of responsibility did not have a significant impact on food waste composting intention, whereas food waste composting intention substantially influenced food waste composting behavior. The results of the multi-group analysis revealed differences in the relationship between awareness of consequences and food waste composting intention across genders and educational levels. This intriguing finding provides new avenues for future research and offers novel insights into the practical applications and promotion of food waste composting. These results will improve the relevant aspects among farmers for eco-friendly farming practices, innovate food waste management strategies, and mitigate environmental deterioration resulting from hazardous agrochemicals. This study expands the current body of literature by providing government regulators and other social enterprises with effective laws, policies, and strategy development guidelines for adopting natural composting on a large scale and enhancing the nutritional value of food to prevent unforeseen health risks caused by toxic chemicals.

## 1 Introduction

The expansion of intensive agriculture has successfully addressed the food needs of the global population [1]. In China, arable land accounts for 9% of the world's population, but feeds 20% of the world's population [2]. Nevertheless, the excessive use of agrochemicals in parallel with technological advancements has led to major health hazards and environmental contamination [3]. China's fertilizer use per hectare is much higher than that of other countries [4]. In line with previous studies, the misuse of chemical fertilizers has adversely impacted ecosystem sustainability through the low soil quality of arable land, increased greenhouse gas emissions, and water contamination [5]. No alternative eco-friendly farming methods have been developed to protect human health and cultivable fields from harmful synthetic inputs [6]. Food waste is the most prevalent form of biowaste globally; food waste [7] could become an environmental burden without proper treatment [8]. Sensible food waste management (effective recycling techniques) proves crucial in preventing ecological damage. For example, making compost from food waste could efficiently recycle garbage and create organic fertilizers with high levels of moisture, nutritional value, and energy content for agribusinesses [9,10].

Composting can transform organic compound waste into useable organic products [11] because one-third (1.3 billion tons) of the food produced annually on a global scale is neglected in the food chain [12]. Despite being a cost-effective and dependable food waste recycling process, technological insufficiency and social issues have hindered its global adoption [10]. China, the country with the world's largest population, has documented an alarming quantity of food waste produced annually in the urban catering industry [13]. The Chinese government has strived to mitigate excessive food waste through specific approaches and regulations. However, optimal food waste recycling and repurposing methods through composting are yet to be established. Despite legislative measures by the local government to encourage farmers to use organic fertilizers, the proportion of Chinese farmers using them in their agricultural production remains alarmingly low [14]. In parallel with previous studies [5], a zero-growth strategy was established for chemical fertilizer manufacturers to promote organic fertilizers for farmers [15]. The complexities underpinning Chinese farmers' reluctance to adopt food waste composting must be addressed, given their low application of ready-made organic fertilizers and the relative novelty of this phenomenon.

Studies involving food waste composting intentions and behavior remain scarce, given the emphasis on farmers' organic fertilizer adoption behavior [16,17]. Thus, the motivation of farmers, as root-level stakeholders in the agricultural industry, to use food waste compost must be thoroughly examined. Factors associated with emotional states, environmental cognition, and beliefs remain underexplored following the empirical focus on farmers' personal traits, pro-environmental behaviors, subjective norms, knowledge, and education [3,5]. Concurrently, further investigations have been proposed by relevant scholars to address the socio-psychological factors affecting farmers' adoption of environment-friendly and non-chemical inputs [18]. Based on empirical analyses derived from past studies, the theory of planned behavior (TPB) and the norm activation model (NAM), which can only justify a small percentage of the variance among the determinants, led to the recommendation of a broad spectrum of theories to explain the intention and adoption of environment-friendly farming [6,19]. Consequently, this study integrated four elements–cognitive, environmental, social, and personal–to evaluate farmers’ FWI and adoption. Under the theory of interpersonal behavior (TIB), this study is one of the first to intensively interview 399 Chinese farmers for pivotal insights into the key determinants of food waste composting adoption as a pro-environmental behavior.

The current study outcomes not only emphasize the significance of China's aim to compost food waste but also offer valuable information to develop effective and long-lasting strategies that facilitate the utilization of large-scale food waste compost. Governments in developing countries should amend their agricultural policies to limit the use of chemical inputs and promote food waste composting. This study could also help nonprofit organizations and social enterprises internalize the primary catalysts of agricultural practices, educate farmers on food waste composting, and enable academics in the agricultural and pro-environmental sectors to consider implementing different determinant types that may influence individual behavioral intentions.

Encouraging farmers to adopt environment-friendly fertilization techniques is critical for achieving sustainable food production [20]. This study emphasizes that composting food waste is an effective alternative to reduce the negative impacts of the overuse of chemical fertilizers and provides valuable information for developing effective and long-lasting strategies that facilitate the utilization of large-scale food waste compost. Currently, the Chinese government lacks active policies to control fertilizer overuse [17]. This study illustrates that governments, in developing countries, should amend their agricultural policies to limit the use of chemical inputs and promote food waste composting. In addition, this study could help nonprofit organizations and social enterprises internalize the primary catalysts of agricultural practices, educate farmers on food waste composting, and enable academics in the agricultural and pro-environment sectors to consider implementing different determinant types that may influence individual behavioral intentions.

## 2 Theory and hypothesis

### 2.1 Theoretical foundation

**Fig. 1:**
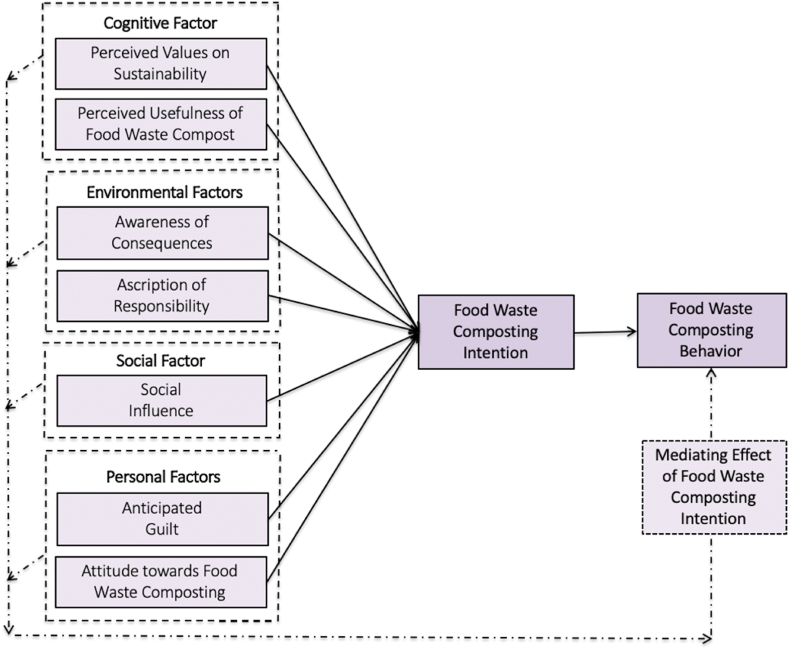
Research framework.

The TPB [6,19,21], NAM [22], and Value-Beliefs-Norms (VBN) theories [23,24] are extensively utilized and well-established in pro-social and pro-environmental behavioral studies. Triandis's [25] TIB signifies personal habits and emotions in developing intentions to be involved in specified behaviors and implies that “intention is an outcome of effect, cognition of consequences, and social and personal norms” [26]. In particular, TIB comprises elements from the TPB, TRA, and VBN to justify the significant impacts of cognitive, environmental, social, and personal factors on one's intention to adopt novel systems, practices, or techniques. The TIB model is primarily applied to examine behaviors in health, public safety, and environmental protection [[26], [27], [28]]. Mumtaz et al. [26] extended the aforementioned model to assess food waste reduction behavior. Khalek and Chakraborty [28] integrated TPB and TIB to develop a model and extended it to assess the factors influencing consumers' willingness to engage in shared consumption. Given the importance of associating TIB with socio-psychological factors related to individual cognitive and emotional factors, organic agriculture is deemed an ecologically sustainable behavior [6]. This study (i) extended TIB as an appropriate theoretical foundation to justify behavioral and decision-making attributes with regard to farmers' food waste composting intention (FWI) and food waste composting behavior (FWB) and (ii) designed and tested a holistic framework (see Fig. 1) by integrating cognitive (perceived value of sustainability and perceived usefulness of food waste compost), environmental (awareness of consequences and ascription of responsibility), social influence, and personal factors (anticipated guilt and attitude towards food waste composting) with TIB as the underpinning theory.

## 3 Hypotheses development

### 3.1 Perceived value on sustainability (PVS)

Values characterize individual decision-making based on various moral standards and social values that require their compliance [29]. Essentially, the desired value criteria can be distinctive and influence people's judgments or behaviors in different circumstances [24]. Riva et al. [30] defined green perceived value as people's evaluation of the benefits of eco-friendly product by comparing what was obtained versus what was spent while considering environmental standards and sustainability expectations, unlike sustainability value. According to Asif et al. [31], individual beliefs imply positive or negative functions of how eco-friendly product values are perceived in the development of adoption intention. People with strong positive values of environmental safety strive to address environmental intricacies and promote eco-social advantages [32]. Individuals' sustainability value-based perceptions favorably affect their personal norms, catalyzing their enthusiasm to engage in sustainability practices [33]. Shin et al. [34] also revealed the significant impact of consumer sustainability values on pro-environmental behaviors for minimal waste production. These discussions lead to the following hypothesis.

H1 The PVS positively affects FWI.

### 3.2 Perceived usefulness of food waste compost (PUS)

Farmers' views of sustainable practices as a means of improving production, soil fertility, food quality, and profitability indicate the perceived usefulness of green agricultural practices [35]. Specifically, farmers may consider using eco-friendly farming products and practices if the associated techniques and biological inputs prove advantageous for higher yields compared to other counterparts [6] and environmental and health protection [36] are achieved. Zhang et al. [37] indicated that farmers may resist cleaner production practices without a holistic comprehension of their usefulness in increasing production and revenue, reducing labor and material expenses, and improving crop survival amidst natural catastrophes. Following Bagheri et al. [38], farmers prioritized biological inputs according to their efficiency in improving the nutritional quality of crops. Empirical studies on farmers’ pro-environmental behavior have identified the significant influence of perceived utility on intention towards sustainable agricultural practices [35], similar to eco-friendly fertilizing technologies [20]. These arguments lead to the following hypothesis.

H2 The PUS positively affects FWI.

### 3.3 Awareness of consequences (AOC)

Notably, AOC characterizes people's perceptions of positive or negative behaviors and action outcomes [39]. Individuals who are highly aware that their activities may have negative implications would change their behavior to avoid such ramifications [24]. Likewise, farmers who realize that disregarding organic fertilizers could induce agricultural non-point source pollution would consciously begin utilizing non-chemical fertilizers to minimize pollution [22]. Nguyen et al. [6] highlighted farmers' rising awareness of the benefits of organic farming and its health and environmental ramifications, prompting the adoption of organic methods. Following Rezaei et al. [19], farmers who are conscious of the positive implications of incorporating non-polluting pest management realize the hazardous environmental effects of chemical pesticides. Such individuals develop strong intentions to engage in eco-friendly pest control techniques. Rastegari Kopaei et al. [40] implied that AOC positively impacted individual attitudes towards composting. These findings lead to the following hypothesis.

H3 The AOC positively impacts FWI.

### 3.4 Ascription of responsibility (AOR)

People's acceptance of accountability for the positive or negative outcomes of prosocial activities or non-activity implies AOR [39]. Fatemi and Rezaei-Moghaddam [41] defined farmers' responsibility in organic farming as the extent to which they are prepared to acknowledge the negative implications of not practicing eco-friendly methods and recognizing their duty to mitigate undesirable environmental influences. Some farmers may prefer utilizing organic fertilizers over their artificial counterparts with a sense of responsibility to safeguard the ecosystem. In Fatemi and Rezaei-Moghaddam [41] and Nguyen et al. [6], farmers' AOR of pro-environmental behavior significantly impacted the adoption intention of organic agricultural activities. Similarly, Xie et al. [22] observed that a stronger AOR among farmers regarding environmental pollution would lead to the adoption of organic fertilizer application. This study, which postulated a similar association between AOR and the intention towards food waste composting among farmers, develop the following hypothesis.

H4 The AOR positively affects FWI

### 3.5 Social influence (SUN)

SUN denotes the communal pressure that impacts individuals' behavior under specific conditions when undertaken with the approval of social peers [42]. Based on Fatemi and Rezaei-Moghaddam's [41] work, SUN, in the context of organic agriculture practices, denotes farmers' perceptions of communal anticipations and pressure to implement pro-environmental practices. Villamayor-Tomas et al. [43] affirmed the positive influence of farmers' recommendations on other farmers' engagement in agro-environmental initiatives. Farmers tend to adopt organic agricultural methods under societal pressure from their community [6]. For example, farmers who observe their neighbors and friends using organic fertilizers acknowledge that they are expected to perform similar adoptions and internalize organic techniques to alleviate reputational harm or social isolation [5]. Rahman et al. [21] and Al Mamun et al. [44] conducted household composting studies and outlined the direct influence of subjective norms, which improved composting intentions following the social pressure of sustaining pro-environmental standards. The following hypothesis is developed based on these facts.

H5 SUN positively affects FWI.

### 3.6 Anticipated guilt (ANG)

Guilt is a negative emotion that catalyzes people to engage in positive actions, such as prosocial behavior [45] to counterbalance their stress, concern, and remorse [46]. Farmers with high environmental awareness may experience guilt if environmental hazards result from harmful agricultural practices [22]. Nguyen et al. [6] reported that farmers who are conscious of the benefits of organic farming feel guilty about the negative environmental impacts caused by toxic chemicals and intend to migrate to organic farming. According to Arkorful et al. [45], people would anticipate feelings of guilt before engaging in pro-environmental behaviors, which is a crucial component in interpreting their pro-environmental behavioral intentions. According to Nguyen et al. [6], farmers demonstrate ethical commitment and intention to incorporate organic agricultural practices for optimal consumer health and protection out of remorse for being negligent previously. These discussions lead to the following hypothesis.

H6 The ANG positively affects FWI.

### 3.7 Attitude towards food waste composting (ATT)

As key determinants of individual intentions [6,19], personal attitudes reflect the degree to which individuals regard actions as acceptable or otherwise [47]. Attitudes towards pro-environmental activities involve people's assessments of environmental protection and conservation practices [48]. Following Fatemi and Rezaei-Moghaddam [41], improving farmers' attitudes towards environmental sustainability increased their involvement in organic agriculture methods. Onwezen et al.‘s [49] study on pro-environmental attitudes emphasized the potential disparities occurring in cross-cultural variances based on the magnitude of the attitude-intention link. Therefore, it is crucial to determine how attitudes influence individual intentions in diverse cultural contexts. Empirically, Al Mamun et al. [48] and Rastegari Kopaei et al. [40] revealed that positive individual attitudes influenced behavioral intentions towards composting. These arguments lead to the following hypothesis.

H7 The ATT positively affects FWI.

### 3.8 Food composting intention (FWI) and food waste composting behavior (FWB)

Behavior implies an intentional action performed following a person's internal judgment of a specified occurrence [50]. The decision to engage in an activity determines whether a specific behavior will flourish [41]. Adopting an ecological activity or product relies on the intention to utilize environmental-friendly products to mitigate negative environmental implications and promote a sustainable lifestyle [[51], [52], [53]]. The term “behavioral intention” in organic farming denotes an individual's desire to eliminate hazardous chemical inputs in agricultural production and adopt organic agricultural practices [44]. The literature gaps between organic practice intentions and behaviors have been highlighted in earlier studies because intentions do not necessarily result in actual implementation [54]. However, Fatemi and Rezaei-Moghaddam [41] and Al Mamun et al. [44] demonstrated a strong and positive link between the intention to adopt organic agriculture and its actual acceptance among farmers. These discussions lead to the following hypothesis.

H8 The FWI positively affects FWB.

All association, hypothesized above, are presented in Fig. 1 below.

## 4 Research methodology

This quantitative cross-sectional study aimed to examine the construct correlations. Partial least squares-structural equation modelling (PLS-SEM), an extensively utilized multivariate and non-parametric method that evaluates path correlations among latent constructs, was performed for statistical analysis using SmartPLS (V.4.0) software [55]. Essentially, PLS-SEM is used in the presence of complex study frameworks and mediating or moderating elements [55]. This study examined the mediating effects of intention towards food waste composting, by incorporating seven independent factors into a sophisticated framework. In summary, PLS-SEM was deemed the most appropriate data analysis method for this study.

### 4.1 Population and sampling

This study employed Chinese farmers of all ages and crop types as the target population. Additionally, the survey emphasized that farmers from second-tier cities with the resources to plant, as opposed to their rural counterparts, seldom dine out. Convenience sampling was used to select readily available respondents from any location and segment [56]. Hair et al. [57] proposed a sample size of 200–400 for the SEM. Meanwhile, the G*power tool employed for minimal sample size computation following Hair et al. [55], revealed a sample size requirement of at least 160 respondents based on the parameters of α err prob = 0.05, effect size = 0.15, power (1-β err prob) = 0.95, and number of predictors = 8. This study gathered data that were three times greater than the specified minimum sample size to mitigate the possible complexities underlying a small sample size.

### 4.2 Data collection

Data were collected from farmers (selected using convenience sampling) attending “Practical Skills Training for New Vocational Farmers” in Zhoukou, Henan Province, China. This training program was held in late spring and early summer (March to June) in 2022, with a total of 20 sessions; over 1000 farmers attended the training program. The data-collection period was limited to May 15 and June 30. Selection of respondents from the participants of the training program for farmers confirmed their prior experience and willingness to learn new methods. The data collection team approached the farmers during and after the training sessions. Complete data were collected from 399 farmers through structured interviews after they signed an informed consent form. The respondents’ personal information was handled with utmost confidentiality. Survey participation was voluntary.

### 4.3 Measurement items

The current study's questionnaire was structured by adapting previously validated questions to meet the research requirements. For example, PVS items were derived from Kim et al. [33] and Han et al. [58], PUS items from Choi et al. [59], AOC, ANG, and FWB items from Attiq et al. [60], AOR items from Kim et al. [61], SUN items from Al Mamun [44], ATT items from Claudy et al. [62] and Gupta and Arora [63], and FWI items from Aktas et al. [64]. This study also employed a 7-point Likert scale to collect data.

## 5 Result

### 5.1 Demographic data

Table 1 presents the demographic characteristics of participants. Specifically, 55.6% of the respondents were female, and the remaining (44.4%) were male. Most individuals (57.6%) were between 18 and 40 years old, whereas the remainder (42.4%) were over 40 years old. Regarding employment, 62.7% of respondents were full-time farmers, 20.0% were self-employed in agriculture-related businesses, and 16% were farmers and occasional short-term workers. A substantial number of respondents (58.1%) had completed higher school education or junior college, whereas the remainder (41.9%) held a Bachelor's degree and above. Most individuals (78.7%) had not received any food waste composting training as opposed to the rest (21.3%). Furthermore, 77.4% of the farmers produced vegetables, compared to the remaining 22.6% who did not.

**Table 1:** Demographic characteristics of the respondents.

	n	%		n	%
*Age*			*Gender*		
18-20	2	0.5	Male	177	44.4
21-30	105	26.3	Female	222	55.6
31-40	123	30.8	Total	399	100.0
41-50	119	29.8	*Family members*		
51-60	46	11.5			
60+	4	1.0	2 and below	57	14.3
Total	399	100.0	3	177	44.4
*Education*			4	132	33.1
			5 and above	33	8.3
High school and below	64	16.0	Total	399	100.0
Junior college	168	42.1	*Frequency of eating out*		
Bachelor's degree	154	38.6			
Master's degree	13	3.3	Hardly	22	5.5
Total	399	100.0	1 to 5 times a month	205	51.4
*Employment*			6 to 10 times a month	92	23.1
			10 to 15 times a month	56	14.0
Farming (Full time)	250	62.7	more than 16 times a month	24	6.0
Farming & occasional short-time work	64	16.0	Total	399	100.0
Self-employment in agriculture-related businesses	80	20.0	*Types of Crops Produced* *Vegetables*		
Retired - Farming Occasionally	5	1.3			
Total	399	100.0	No	90	22.6
*Monthly expenditure on food*			Yes	309	77.4
			Total	399	100.0
less than RMB 2000	25	6.3	*Fruits*		
RMB 2001–3000	103	25.8			
RMB 3001–4000	136	34.1	No	347	87.0
RMB 4001–5000	74	18.5	Yes	52	13.0
RMB 5001–6000	33	8.3	Total	399	100.0
above RMB 6001	28	7.0	*Food crops*		
Total	399	100.0			
*Received training*			No food crops	294	73.7
			Yes	105	26.3
Yes	85	21.3	Total	399	100.0
No	314	78.7	*Other cash crops*		
Total	399	100.0			
*Number of times crops planted last year*			No	357	89.5
			Yes	42	10.5
0∼2 times	309	77.4	Total	399	100.0
3∼5	43	10.8	*Mixed planting*		
6∼8	22	5.5			
9∼11	12	3.0	No	335	84.0
more than 11	13	3.3	Yes	64	16.0
Total	399	100.0	Total	399	100.0

### 5.2 Common method bias

Harman's single-factor test, a well-established means of confirming that the research model is not significantly impacted by CMB [65], demonstrated that the single component only accounted for 35.669% of the variance, compared with Podsakoff et al.‘s [66] highest cut-off of 50%. As such, the CMB did not hamper the current work. Moreover, based on Kock [67], all variance inflation factor (VIF) values must be less than the highest cutoff of 3.3 to ensure CMB from single-source data. The VIF values (Table 2), ranging from 1.093 to 2.487, fulfilled the threshold criteria.

**Table 2:** VIF values for all variables obtained using Full Collinearity Test.

Variables	Variance Inflation Factor Values
Perceived Values on Sustainability	1.093
Perceived Usefulness of Food Waste Compost	1.157
Awareness of Consequences	1.221
Ascription of Responsibility	1.385
Social Influence	1.508
Anticipated Guilt	1.492
Attitude towards Food Waste Composting	1.505
Food Waste Composting Intention	1.616
Food Waste Composting Behavior	2.487

### 5.3 Multivariate normality

Web Power, a statistical web tool, was employed in this study to assess multivariate skewness and kurtosis to determine multivariate normality issues. The study data were not normally distributed, as both multivariate kurtosis and skewness *p*-values were <0.05 [68]]. Therefore, this study used PLS-SEM for data analysis.

### 5.4 Measurement model (outer model)

Hair et al. [55] proposed that the measurement model must be assessed for pre-structural model assessment. The outer model was evaluated for internal consistency, reliability, convergent validity, and discriminant validity to validate the robustness of the measurement model.

### 5.5 Internal consistency and convergent validity

Dijkstra-Hensele's *rho* (*rho_A*), Cronbach's alpha (CA), average variance extracted (AVE), and composite reliability (CR), which assessed the internal consistency and convergent validity of the research constructs, indicated strong internal consistency and reliability with *rho_A*, CA, and CR values exceeding 0.70 [69]. In Table 3, all study constructs demonstrated CA (from 0.959 to 0.976), *rho_A* (from 0.963 to 0.978), and CR (from 0.969 to 0.981) values exceeding the 0.7 thresholds. The study outcomes validated the high reliability and internal consistency of the model. Notably, AVE analysis primarily aimed to assess convergent validity by estimating the proportion of construct variation that could be explained by the latent components [55]. Following Hair et al. [55], AVE values should exceed 0.5 to ascertain that the model and its components denote strong convergent validity. Based on the AVE values presented in Table 3 (0.860–0.912), the values fulfilled the threshold criteria and implied strong convergence validity.

**Table 3:** Validity and reliability of variables.

Variables	Mean	Standard Deviation	Cronbach's Alpha	Dijkstra-Hensele's *rho*	Composite Reliability	Average Variance Extracted
Perceived values on sustainability (PVS)	4.7519	1.81952	0.972	0.978	0.978	0.901
Perceived usefulness of food waste compost (PUS)	4.4556	1.67026	0.959	0.963	0.969	0.860
Awareness of Consequences (AOC)	4.6341	1.57028	0.965	0.965	0.973	0.876
Ascription of Responsibility (AOR)	4.6266	1.63517	0.968	0.970	0.975	0.888
Social Influence (SUN)	4.5784	1.68232	0.976	0.978	0.981	0.912
Anticipated Guilt (ANG)	4.5253	1.62689	0.968	0.971	0.975	0.885
Attitude towards food waste composting (ATT)	4.6732	1.61066	0.973	0.974	0.979	0.904
Food Waste Composting Intention (FWI)	4.6095	1.59868	0.970	0.971	0.977	0.893
Food Waste Composting Behavior (FWB)	4.7223	1.48854	0.969	0.970	0.976	0.890

### 5.6 Discriminant validity

Cross-loadings, the Heterotrait-Monotrait (HTMT) ratio, and the Fornell-Larcker criteria have been extensively applied to evaluate model discriminant validity. The square root of the AVE of a construct should exceed all other latent variables in the row and column that it contains when applying the Fornell-Larcker criteria [55]. Table 4 presents the Fornell-Larcker criterion values for each construct. Consequently, these values exceeded any correlations in the pertinent columns and rows, including the respective constructs. Strong discriminant validity was evident when the HTMT values for all constructs remained below 0.85 [70]. In line with Table 4, all HTMT values of the components are below 0.85, which confirms discriminant validity.

**Table 4:** Discriminant Validity using Fornell-Larcker criteria and Heterotrait-Monotrait ratio.

	PVS	PUS	AOC	AOR	SUN	ANG	ATT	FWI	FWB
*Fornell-Larcker Criterion*									
PVS	0.949								
PUS	0.134	0.928							
AOC	0.107	0.176	0.936						
AOR	0.168	0.230	0.257	0.942					
SUN	0.190	0.239	0.261	0.403	0.955				
ANG	0.091	0.226	0.230	0.314	0.373	0.941			
ATT	0.116	0.196	0.288	0.351	0.411	0.402	0.951		
FWI	0.158	0.258	0.334	0.285	0.352	0.449	0.377	0.945	
FWB	0.272	0.350	0.385	0.482	0.530	0.519	0.532	0.578	0.943
*Heterotrait-Monotrait Ratio (HTMT)*									
PVS									
PUS	0.138								
AOC	0.110	0.181							
AOR	0.174	0.238	0.265						
SUN	0.192	0.245	0.269	0.414					
ANG	0.092	0.233	0.237	0.323	0.382				
ATT	0.118	0.203	0.297	0.361	0.422	0.413			
FWI	0.161	0.267	0.345	0.294	0.361	0.462	0.387		
FWB	0.279	0.361	0.398	0.497	0.544	0.534	0.548	0.595	

**Note:** PVS: Perceived Value of Sustainability; PUS: Perceived Usefulness of Food Waste Compost; AOC: Awareness of Consequences; AOR: Ascription of Responsibility; SUN: Social Influence; ANG: Anticipated Guilt; ATT: Attitude Towards Food Waste Composting; FWI: Food Waste Composting Intention; FWB: Food Waste Composting Behavior.

**Fig. 2:**
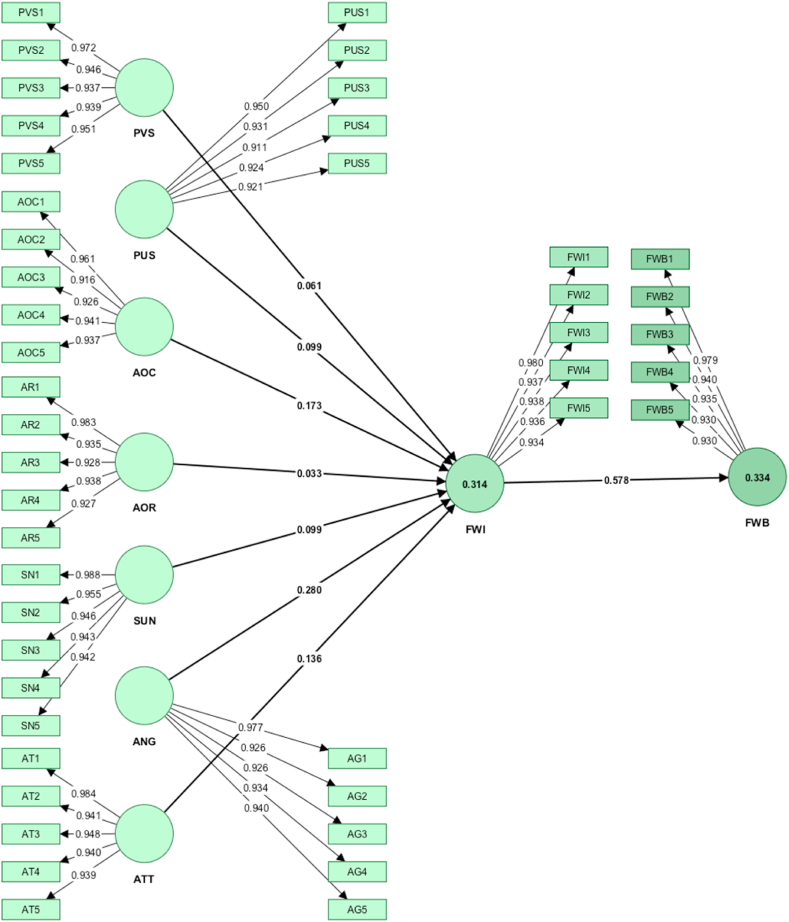
Measurement model with output.

Chin et al. [71] proposed that all factor loadings should exceed 0.70, and all construct factor loadings, which ranged from 0.916 to 0.988, exceeded the recommended threshold, as presented (in bold font) in Table 5 and Fig. 2. Consequently, the three validity tests employed in this study presented evidence of strong discriminant validity within the constructs.

**Table 5:** Discriminant Validity using Loading and Cross-Loading Values.

CODE	PVS	PUS	AOC	AOR	SUN	ANG	ATT	FWI	FWB
PVS1	**0.972**	0.112	0.105	0.163	0.196	0.102	0.113	0.151	0.277
PVS2	**0.946**	0.134	0.086	0.164	0.154	0.079	0.090	0.123	0.229
PVS3	**0.937**	0.126	0.104	0.143	0.190	0.058	0.116	0.160	0.267
PVS4	**0.939**	0.115	0.106	0.176	0.169	0.096	0.103	0.147	0.257
PVS5	**0.951**	0.145	0.106	0.155	0.184	0.097	0.124	0.162	0.257
PUS1	0.131	**0.950**	0.164	0.208	0.227	0.198	0.175	0.238	0.328
PUS2	0.129	**0.931**	0.194	0.209	0.242	0.213	0.174	0.265	0.351
PUS3	0.108	**0.911**	0.117	0.156	0.168	0.196	0.175	0.219	0.295
PUS4	0.121	**0.924**	0.173	0.233	0.252	0.219	0.181	0.242	0.316
PUS5	0.129	**0.921**	0.159	0.257	0.213	0.222	0.207	0.228	0.327
AOC1	0.114	0.174	**0.961**	0.233	0.250	0.204	0.255	0.295	0.366
AOC2	0.126	0.197	**0.916**	0.259	0.249	0.208	0.262	0.320	0.333
AOC3	0.097	0.173	**0.926**	0.257	0.274	0.252	0.295	0.302	0.390
AOC4	0.098	0.153	**0.941**	0.221	0.228	0.229	0.261	0.326	0.368
AOC5	0.069	0.126	**0.937**	0.233	0.223	0.183	0.275	0.318	0.345
AOR1	0.159	0.236	0.248	**0.983**	0.399	0.290	0.348	0.276	0.477
AOR2	0.159	0.197	0.266	**0.935**	0.377	0.327	0.335	0.277	0.454
AOR3	0.140	0.208	0.225	**0.928**	0.374	0.290	0.295	0.258	0.425
AOR4	0.161	0.217	0.261	**0.938**	0.359	0.303	0.325	0.282	0.457
AOR5	0.175	0.224	0.206	**0.927**	0.390	0.268	0.348	0.249	0.454
SUN1	0.199	0.224	0.249	0.395	**0.988**	0.357	0.409	0.342	0.517
SUN2	0.185	0.224	0.224	0.373	**0.955**	0.350	0.379	0.337	0.498
SUN3	0.206	0.252	0.255	0.402	**0.946**	0.353	0.400	0.344	0.526
SUN4	0.138	0.206	0.248	0.357	**0.943**	0.339	0.386	0.296	0.468
SUN5	0.171	0.232	0.270	0.391	**0.942**	0.378	0.388	0.356	0.514
ANG1	0.090	0.216	0.220	0.318	0.376	**0.977**	0.411	0.434	0.514
ANG2	0.080	0.214	0.226	0.280	0.305	**0.926**	0.351	0.419	0.467
ANG3	0.050	0.208	0.219	0.253	0.321	**0.926**	0.331	0.383	0.450
ANG4	0.093	0.178	0.183	0.314	0.369	**0.934**	0.399	0.403	0.495
ANG5	0.110	0.243	0.232	0.310	0.378	**0.940**	0.395	0.465	0.508
ATT1	0.098	0.185	0.282	0.335	0.401	0.385	**0.984**	0.370	0.521
ATT2	0.137	0.208	0.274	0.340	0.390	0.397	**0.941**	0.364	0.507
ATT3	0.065	0.164	0.267	0.334	0.383	0.389	**0.948**	0.344	0.496
ATT4	0.098	0.191	0.281	0.318	0.372	0.352	**0.940**	0.345	0.484
ATT5	0.151	0.184	0.266	0.339	0.408	0.388	**0.939**	0.365	0.519
FWI1	0.155	0.244	0.312	0.280	0.344	0.438	0.369	**0.980**	0.563
FWI2	0.192	0.248	0.299	0.280	0.341	0.411	0.350	**0.937**	0.567
FWI3	0.139	0.231	0.326	0.262	0.331	0.448	0.340	**0.938**	0.537
FWI4	0.116	0.261	0.325	0.273	0.310	0.404	0.328	**0.936**	0.533
FWI5	0.144	0.237	0.318	0.252	0.338	0.420	0.394	**0.934**	0.529
FWB1	0.279	0.346	0.379	0.456	0.508	0.506	0.511	0.571	**0.979**
FWB2	0.262	0.335	0.359	0.475	0.502	0.492	0.484	0.567	**0.940**
FWB3	0.266	0.318	0.362	0.459	0.483	0.469	0.501	0.519	**0.935**
FWB4	0.265	0.331	0.351	0.429	0.500	0.480	0.496	0.533	**0.930**
FWB5	0.212	0.316	0.363	0.451	0.506	0.497	0.518	0.532	**0.930**

**Note:** PVS: Perceived Value of Sustainability; PUS: Perceived Usefulness of Food Waste Compost; AOC: Awareness of Consequences; AOR: Ascription of Responsibility; SUN: Social Influence; ANG: Anticipated Guilt; ATT: Attitude Towards Food Waste Composting; FWI: Food Waste Composting Intention; FWB: Food Waste Composting Behavior.

### 5.7 Structural model (inner model)

Hair et al. [55] proposed the use of path coefficients (beta values-β), coefficient of determination (*R*^*2*^), and effect size (*f*^*2*^) for structural model assessment. The p-values, t-values, and path coefficients for each correlation were calculated using a bootstrapping approach for hypothesis testing. The indirect effects were then analyzed to identify the mediating effect.

### 5.8 Hypothesis testing

Table 6 presents the hypothesis testing outcomes after bootstrapping. First, the PVS-FWI relationship reported a positive β-value of 0.061 and a moderate t-value of 1.367, albeit with a p-value of 0.086, which was statistically insignificant and did not support H1. The positive PUS-FWI association supported H2. This assumption was confirmed by the bootstrapping results, which showed statistically significant values (β = 0.099, t = 2.089, p < 0.05). 10.13039/100014337Furthermore, AOC significantly and positively impacted FWI with β = 0.173, t = 3.403, p < 0.001 and supported H3. The AOR-FWI association demonstrated no statistically significant p-value (0.270) despite denoting a positive β-value (0.033), which did not support H4. In contrast, FWI was significantly impacted by 10.13039/501100004296SUN (β = 0.099, t = 1.667, p < 0.05), ANG (β = 0.280, t = 5.100, p < 0.001), and ATT (β = 0.136, t = 2.426, p < 0.001), with positive β-values and significant t- and p-values, thereby supporting H5, H6, and H7. Finally, FWI significantly affected FWB with statistically significant values of (β = 0.578, t = 12.896, p < 0.001), thereby supporting H8.

**Table 6:** Hypothesis testing.

Hypo	Direct Effects	CI-Min	CI-Max	Beta	*t* *value*	*p* *value*	Decision
H_1_	PVS → FWI	−0.015	0.133	0.061	1.367	0.086	Rejected
H_2_	PUS → FWI	0.022	0.176	0.099	2.089	0.018	Supported
H_3_	AOC → FWI	0.090	0.257	0.173	3.403	0.000	Supported
H_4_	AOR → FWI	−0.054	0.122	0.033	0.612	0.270	Rejected
H_5_	SUN → FWI	0.004	0.198	0.099	1.667	0.048	Supported
H_6_	ANG → FWI	0.189	0.372	0.280	5.100	0.000	Supported
H_7_	ATT → FWI	0.045	0.228	0.136	2.426	0.008	Supported
H_8_	FWI → FWB	0.500	0.647	0.578	12.896	0.000	Supported

**Note:** PVS: Perceived Value of Sustainability; PUS: Perceived Usefulness of Food Waste Compost; AOC: Awareness of Consequences; AOR: Ascription of Responsibility; SUN: Social Influence; ANG: Anticipated Guilt; ATT: Attitude Towards Food Waste Composting; FWI: Food Waste Composting Intention; FWB: Food Waste Composting Behavior.

#### 5.8.1 The coefficient of determination (R^2^)

The degree of explained variance (*R*^*2*^) is the amount of variance that a linear model can explain in terms of the dependent variable variation. Endogenous latent variables with *R*^*2*^ values of >0.75, >0.50, or =>0.25 are classified as considerable, moderate, or weak, respectively [69]. Table 7 presents the construct *R*^*2*^ values. In line with the analyses, PVS, PUS, AOC, AOR, SUN, ANG, and ATT explained only 30.1% of the variance in the FWI, indicating low explanatory power. Similarly, the *R*^*2*^ value for FWB (0.332) implied a low explanatory power of FWI, with 33.2% of the variance explained by FWB.

**Table 7:** Coefficient of determination.

Variables	R Square	R Square Adjusted	Explanatory power
Food waste composting Intention	0.314	0.301	Weak
Food waste composting Behavior	0.334	0.332	Weak

**Note:** R^2^ value interpretation (≥0.75- Significant,≥0.50- moderate,≥0.25- Weak) [69].

#### 5.8.2 The effect size (f^2^)

The effect size (*f*^*2*^) assesses the significant impact of exogenous variables on endogenous constructs based on the specific variance of exogenous components rather than their combined variance [69]. In Cohen's study [72], the effects were classified as trivial (<0.02), minor (≥0.02), medium (=0.15), or major (=0.35). Nevertheless, it may prove challenging to determine the appropriateness of general guidelines to attain a significant impact size, given the significant differences in framework attributes and study domains [57]. As shown in Table 8, the outcomes derived from the effect size assessment reflect a small effect for all associations, excluding FWI and FWB. Effect sizes ranged from 0.001 to 0.087. FWI denoted a major effect with an effect size of 0.501.

**Table 8:** Effect size of the variables.

	Food Waste Composting Intention	Food Waste Composting Behavior
Perceived values on sustainability	0.005	–
Perceived usefulness of food waste compost	0.013	–
Awareness of Consequences	0.038	–
Ascription of Responsibility	0.001	–
Social Influence	0.010	–
Anticipated Guilt	0.087	–
Attitude towards food waste composting	0.019	–
Food Waste Composting Intention	–	0.501

**Note:** f^2^ score interpretation (≥0.35- substantial effect size,≥0.15– medium effect size,≥0.02- small effect size and <0.02- trivial effect size) (Cohen, 2013).

#### 5.8.3 Indirect effect (mediation)

In addition to assessing direct correlations across variables, Hair et al. [69] highlighted the need to incorporate the indirect effects of all associations to gain a sound understanding of construct effects. Table 9 presents the mediation (indirect) effects of FWI. Resultantly, FWI fully mediated the associations of PUS to FWB (β = 0.057, t = 2.032, p < 0.05), AOC to FWB (β = 0.100, t = 3.309, p < 0.001), ANG to FWB (β = 0.162, t = 4.602, p < 0.001), and ATT to FWB (β = 0.079, t = 2.329, p < 0.05). For the three remaining associations, FWI reflected no mediation impact and statistically significant p-values for PVS to FWB (p > 0.05), AOR to FWB (p > 0.05), and SUN to FWB (p > 0.05).

**Table 9:** Indirect effects of food waste composting intention.

	Beta	*t-Value*	*p-value*	Decision
PVS → FWI → FWB	0.035	1.349	0.089	No Mediation
PUS → FWI → FWB	0.057	2.032	0.021	Mediation
AOC → FWI → FWB	0.100	3.309	0.000	Mediation
AOR → FWI → FWB	0.019	0.604	0.273	No Mediation
SUN → FWI → FWB	0.057	1.614	0.053	No Mediation
ANG → FWI → FWB	0.162	4.602	0.000	Mediation
ATT → FWI → FWB	0.079	2.329	0.010	Mediation

**Note:** PVS: Perceived Value of Sustainability; PUS: Perceived Usefulness of Food Waste Compost; AOC: Awareness of Consequences; AOR: Ascription of Responsibility; SUN: Social Influence; ANG: Anticipated Guilt; ATT: Attitude Towards Food Waste Composting; FWI: Food Waste Composting Intention; FWB: Food Waste Composting Behavior.

### 5.9 Multi-group analysis

The study model was evaluated using a multi-group analysis (MGA) for in-depth evaluation and interpretation. As an optimal approach for examining subgroup heterogeneity, PLS-MGA is suitable for assessing moderation across multiple (rather than single) associations [55]. Measurement invariance was first validated to confirm the validity of the effects before applying PLS-MGA. Specifically, the measurement invariance of composite models (MICOM) approach was used to assess the degree of homogeneity between groups.

Five categories of demographic details were considered for the PLS-MGA. Each category contained two groups as follows: Category 1 (Gender: male vs. female); Category 2 (Age: below 40 vs. above 40); Category 3 (Education: junior college and below vs. bachelor's degree and above); Category 4 (Training Received: no vs. yes; (Category 5. Produced Vegetables: no vs. yes). All categories were first evaluated for MGA variations. The permutation p-values of all constructs exceeded 0.05, thereby confirming the measurement invariances among the analyzed groups. Consequently, the study commenced by examining the path coefficient differences and p-values using PLS-MGA. Based on the study outcomes, the p-values for categories and all groups exceeded 0.05, except for the effect of awareness of consequences on food waste composting intention between gender and education groups (see Table 10). The results of the multi-group analysis indicated significant differences in the relationship between AOC and FWI across gender and education groups.

**Table 10:** Multi-group analysis.

Groups →	Male - Female		Age Below 40 – Age Above 40		Junior college and below - Bachelor degree and above		(No) Training - (Yes) Training		(No) Vegetables - (Yes) Vegetables	
Associations ↓	β-Diff	*p Value*	β-Diff	*p Value*	β-Diff	*p Value*	β-Diff	*p Value*	β-Diff	*p Value*
PVS→FWI	−0.019	0.418	−0.045	0.314	0.125	0.084	0.100	0.229	−0.010	0.473
PUS→FWI	0.003	0.487	−0.043	0.323	−0.024	0.399	−0.031	0.405	0.144	0.111
AOC→FWI	−0.056	0.288	−0.021	0.426	−0.046	0.320	0.033	0.408	−0.236	0.066
AOR→FWI	0.193	0.032	−0.008	0.472	−0.293	0.003	−0.120	0.198	0.071	0.297
SUN→FWI	−0.174	0.067	0.079	0.274	0.128	0.131	0.000	0.492	0.043	0.389
ANG→FWI	−0.037	0.372	−0.018	0.441	−0.064	0.269	0.114	0.207	0.031	0.425
ATT→FWI	−0.177	0.058	0.147	0.109	0.037	0.365	0.077	0.284	−0.028	0.416
FWI→FWB	−0.046	0.310	−0.018	0.418	0.055	0.278	0.055	0.322	0.027	0.396

**Note:** PVS: Perceived Value of Sustainability; PUS: Perceived Usefulness of Food Waste Compost; AOC: Awareness of Consequences; AOR: Ascription of Responsibility; SUN: Social Influence; ANG: Anticipated Guilt; ATT: Attitude Towards Food Waste Composting; FWI: Food Waste Composting Intention; FWB: Food Waste Composting Behavior.

## 6 Discussions

The implications of PVS, PUS, AOC, AOR, SUN, ANG, and ATT were examined. Furthermore, the effect of intention on the actual use of food waste compost was assessed. All study correlations in the framework were positive and significant following the PLS-SEM analysis, excluding the effect of AOR on intention. The empirical outcomes are compared or justified with past literature in the subsequent paragraphs, along with potential reasons, interpretations, and indications.

Farmers' intention to compost food waste was not affected by PVS. This result contradicts Li and Wu's [5] recent research on organic fertilizer use. Farmers remain relatively unaware of the necessity of preserving ecological harmony and increasing agricultural production, following their focus on maximizing financial profits through large-scale production through chemical inputs. In other words, farmers possess insufficient knowledge that their farmlands may soon turn infertile due to ecological imbalances resulting from chemical inputs, which can only be addressed through natural compost.

Essentially, PUS was significantly associated with the respondents’ intentions, in line with previous research [20]. Farmers have come to accept and utilize food waste compost following their realization of its multilevel benefits, including disease prevention, pest control, low water and soil contamination, high nutritional value of food, preservation of plant and animal biodiversity, and protection of farmers and public health, as opposed to chemical fertilizers (soil fertility). Contemporary farmers have also come to perceive natural fertilizers as more yield-generating and profitable than their synthetic counterparts.

AOC positively influenced farmers' food composting intentions, following Rezaei et al.‘s [19] study on farmers' eco-friendly pest management practices. Farmers currently encountering issues of excessive hazardous chemical inputs are cognizant of the different complexities that may arise without using eco-friendly fertilizers, such as low food quality and chronic ailments among consumers) without using eco-friendly fertilizers. Furthermore, these individuals struggle with soil infertility and the rapid loss of cultivable lands owing to the rapid growth of chemical factories and their uncontrolled chemical waste discharged into water sources and open fields. Such adversities have rendered them more empathetic and concerned about the negative implications of chemical fertilizers.

Intriguingly, respondents’ (farmers) intentions to compost food waste were not significantly influenced by their sense of accountability. This outcome contradicts recent research on organic farming practices [6,22] and indicates an alarmingly low level of food waste composting adoption among root-level farmers. Many farmers who focus solely on using chemical inputs for profitability remain uncertain about their contribution to environmental sustainability. Moreover, farmers have been misled into thinking that corrective measures to prevent environmental damage are the responsibility of the government and legislative authorities.

Concurrently, SUN considerably affected intention, according to Al Mamun et al. [48] and Rahman et al. [21]. Perceivably, the popularity of food waste composting among farmers who have benefitted from the practice has led them to influence their peers. These individuals can conveniently recommend this practice to their communities in the absence of financial risk. Environmental protection has become a prevalent social trend among farmers, leading to the adoption of eco-friendly farming practices and the prevention of blame, embarrassment, and social isolation.

The current study's findings, which revealed a significant and positive association between ANG and the intention to compost food waste, corresponded to those of Han et al. [58] and Onwezen [49] in terms of eco-friendly consumption. Farmers are aware of the negative implications of chemical fertilizers and experience moral remorse about causing an ecosystem imbalance, following their emphasis on high profitability. Predictably, remorseful farmers who regret supplying foods with poor nutrient values owing to chemical inputs have a negative effect on consumer health.

Meanwhile, ATT positively influenced farmers' intentions to use these composts, following the research of Al Mamun et al. [48] and Rastegari Kopaei et al. [40] on green and household composting. Justifiably, farmers' mental preparedness to positively perceive food-waste composting technologies increased their accessibility. Farmers’ positive ATT could also have improved following their view of food waste compost as an affordable, eco-friendly, and nutrition-rich organic manure.

The actual use of food waste compost was enhanced by the intention to compost food waste, based on Fatemi and Rezaei-Moghaddam's [41] and Rezaei et al.‘s [19] study on organic agriculture practices. Farmers can utilize food waste composting by setting goals. Additionally, the severity of environmental deterioration and disasters necessitates alternative eco-friendly agricultural inputs, thus inspiring farmers to generate compost from food waste. Under the current ecological circumstances, food waste composting has become a necessity rather than a choice.

Lastly, the results of the multi-group analysis indicated differences in the relationship between awareness of consequences and food waste composting intention across different gender groups. This study speculates that one possible reason for this result is that, in Chinese families, homemaking duties are typically performed by female, who are more frequently exposed to kitchen waste in their daily lives, compared to male. This may contribute to female being more sensitive to the reuse of kitchen waste than male. This speculation aligns with previous research. In a study on advertising marketing effects, Meyers-Levy and Loken [73] suggested that frequent exposure to information about a particular object leads to increased sensitivity to that object by the information receiver. In the multi-group analysis, the sample was divided into two groups based on different levels of education. One group consisted of individuals without college education (junior college and below; 58.1%), whereas the other group consisted of those with a bachelor's degree or above. In China, traditional farmers and owners of small-scale farmlands generally do not have a bachelor's degree level of education, whereas some new farmers or farm managers tend to have higher levels of education. Previous studies have shown that people with higher levels of education are more concerned about chemical pollution resulting from human activities [74]. However, the findings of this study reveal the interest of most traditional Chinese farmers in composting food waste. This could be attributed to the relative accessibility and lower cost of organic fertilizers compared to chemical fertilizers or perhaps to the high prices of organic food. This was a fascinating and practically significant discovery.

## 7 Implications

### 7.1 Theoretical implications

This study expands the current body of knowledge on eco-friendly agricultural practices by thoroughly examining real product growers, a demographic segment that remains underexplored in the context of food waste composting. There is a paucity of research identified on the use of food waste compost in large-scale agricultural resilience, despite much local research on organic farming methods and food waste composting intentions among individual households. In examining farmers' intentions towards organic agricultural practices, most studies have used TPB [19,21] or TPB-NAM integration [6,19] as the underpinning theory while disregarding emotional states, perceptions of consequences, and perceived usefulness. Consequently, this study applies TIB and boosts it with three additional factors (PVS, PUS, and ANG) to address the gaps in the existing literature. Intriguingly, the current study is one of the first to employ TIB and integrate them with several novel components for food waste composting. The aforementioned elements were statistically significant and reflected positive influences on farmers' composting food waste intentions, implying the robustness of the proposed framework. It is crucial to address various socio-physiological, personal-emotional, and environmental cognition aspects, as food waste compost may be regarded as a low-yielding fertilizer compared to its chemical counterpart, which inevitably evokes ecological sustainability concerns. As such, this study aimed to cover a broad range of variables from multiple aspects that affect farmers' inclination to use food waste compost. The inclusion of AOC and AOR (two key components of the VBN model) in this framework implies the significance of integrating and rearranging components from many other theoretical models to examine farmers’ pro-environmental behaviors across multiple circumstances.

### 7.2 Practical implications

Practically, the current work significantly impacts the existing agricultural practices and economic progress. For example, this study effectively expanded the commercialization of food waste composting to save farmers' material and operational costs and conserve the environment by eliminating the use of toxic fertilizers and chemicals. Large-scale food waste composting and its subsequent use in extensive agricultural fields remain a relatively novel phenomenon in China, given the low exposure to its economic benefits. The empirical outcomes derived from this study could facilitate government bodies, NGOs, and other social enterprises to mitigate this major barrier and emphasize key factors while encouraging the farming community to recycle food waste by composting. In this regard, the factors and perspectives considered in this study complement the development of food waste composting and other eco-friendly farming practices to resolve the issues underpinning food waste management. Several practical recommendations on the factors influencing Chinese farmers' intentions towards composting food waste are highlighted in the following section in line with the study's findings.

Farmers must be highly sensitive to environmental sustainability as a matter of human control, based on the weak influence of the PVS highlighted in this research. Agricultural departments and other social enterprises should collaborate to communicate accurate information on eco-friendly farming technologies, provide long-term training on food waste composting techniques to farmers, and enhance their awareness. Given the significant influence of PUS, agricultural specialists and scientists must proactively develop novel and viable composting techniques and machinery for farmers’ rapid learning in terms of usage and compatibility compared with conventional chemical inputs. Stakeholders in public environmental management and agricultural development departments and other non-profit companies should strive to educate farmers of all groups on (i) the benefits of composting food waste and (ii) the adverse implications of synthetic fertilizers on the environment, as the AOC proved significant in this study.

Policy enforcers should systematically develop communal gatherings, media campaigns, and documentary showcase programs on food waste composting to improve farmers' levels of awareness. Waste management companies should promote cost-effective food waste composting to restore soil fertility and produce affordable and nutrition-rich fertilizers. As the AOR does not significantly affect intention, this study highlights the need for government bodies and policymakers to take prompt measures and implement effective solutions. For example, government authorities can enforce laws that limit the use of chemical inputs to inculcate a sense of accountability among farmers or increase the price of chemical fertilizers to encourage farmers to use alternative and cost-effective methods, such as food waste compost. The Department of Agriculture can also publish soil test results and reports on soil quality deterioration following excessive chemical inputs to develop a sense of accountability among farmers in adopting food waste composts. In this study, empirical outcomes on the significant and beneficial effects of SUN highlight the value of extensive advertising, information sharing, and the promotion of other farmers’ success stories to the farmer community at large. Furthermore, considering the results of the multi-group analysis and the positive influence of social influence on food waste composting intention, a more practical recommendation might be to employ appropriate marketing strategies or hire people with higher levels of education for promotion purposes. As ANG was represented as the primary catalyst in forming an intention, information sharing on the difficulties and losses encountered by pollution-affected farmers due to chemical inputs could enhance feelings of guilt among other farmers for not substituting synthetic inputs with natural compost in their fields. Relevant legislators and government authorities should enforce laws with heavy penalties to prevent ecological damage and make farmers feel guilty about using chemical fertilizers and disrupting their natural ecological balance.

## 8 Conclusion

Given the complexities underpinning food waste recycling and management in the wake of global sustainability, this study aimed to identify the key determinants that encourage farmers to adopt food waste compost rather than employing chemical fertilizers. The impacts and correlations of socio-physiological, personal-emotional, environmental, and cognitive factors on Chinese farmers were evaluated using TIB as the underlying theory and three additional constructs. The research respondents, comprising 399 farmers across multiple regions of China, were interviewed face-to-face using a structured survey. Based on the statistical analysis, PVS, PUS, AOC, SUN, ANG, and ATT significantly and positively impacts respondents’ intentions to compost food waste. Surprisingly, the insignificant influence of AOR on intention implies that farmers are yet to take responsibility for the environmental damage caused by chemical inputs. The novelty of food waste composting and the misinterpretation of this alternative as a low-yielding method in China have failed to convince farmers to cease using chemical fertilizers. However, the results of the multi-group analysis indicates that the relationship between awareness of consequences and food waste composting intention varies across different levels of education and gender. Thus, critical components that can (i) create optimal strategies and policies in China and other emerging nations, (ii) support eco-friendly agriculture developments, and (ii) foster a responsive system to promote food waste compost adoption arere highlighted in the study outcomes. Such findings could facilitate the establishment of novel laws and regulations that require the use of organic rather than artificial fertilizers for improved soil fertility, alleviation of issues associated with food waste disposal, a sustainable ecosystem, and economic growth.

This study had several limitations that should be addressed in future studies. First, the use of conventional sampling for data collection purposes rendered it challenging for outcome generalization to occur. Further research should include large sample sizes derived from other areas and demographic groups to improve the generalizability of the model and knowledge of the composting process. Second, only a few variables associated with ATT and intention were examined in this study, which could have inadvertently neglected crucial elements such as government support and subsidies. Scholars should consider more constructs to provide a sound understanding of the domain of the study. If future research aims to incorporate more practically significant factors, this study suggests utilizing a Fuzzy-set Qualitative Comparative Analysis (fsQCA) in conjunction with both quantitative and qualitative analyses. Prior to designing the research framework, conducting in-depth interviews on a small scale can provide insights into potential latent variables.

## Ethical approval

Human Research Ethics Committee of Changzhi University approved this study (CZ-2022-0049).

## Funding

This research was funded by UKM - Graduate School of Business, 10.13039/501100004515Universiti Kebangsaan Malaysia (Grant number: GSB-2023-007).

Item-group: IG000074 (Editor article).

## Author contribution statement

Abdullah Al Mamun: Performed the experiments; Analyzed and interpreted the data; Contributed reagents, materials, analysis tools or data; Wrote the paper. Qing Yang; Farzana Naznen; Norzalita Abd Aziz: Conceived and designed the experiments; Performed the experiments; Wrote the paper. Muhammad Mehedi Masud: Conceived and designed the experiments; Analyzed and interpreted the data; Wrote the paper.

## Data availability statement

Data included in article/supplementary material/referenced in article.

## Declaration of competing interest

The authors declare the following financial interests/personal relationships which may be considered as potential competing interests: Abdullah Al Mamun reports financial support was provided by 10.13039/501100004515UKM - Graduate School of Business, Universiti Kebangsaan Malaysia. Corresponding author is the associate editor of the journal (Heliyon).
