# Supplementary material for: Modelling the mass adoption potential of food waste composting among rural Chinese farmers
Source: Heliyon. 2023 Aug 9;9(8):e18998. doi: 10.1016/j.heliyon.2023.e18998 (PMC10440538; doi:10.1016/j.heliyon.2023.e18998)
Supplement: Multimedia component 1 [file mmc1.docx]

**Supporting Material 1.** Survey Instrument

| Code | Items |
| --- | --- |
| PVS1 | I feel that it is critical to live in harmony with other animals and nature by implementing sustainable production and consumption practices. |
| PVS2 | Sustainable production and consumption, in my opinion, is the best way to safeguard natural resources. |
| PVS3 | I feel that household sustainability measures (e.g., minimizing food, water, electricity, natural gas, and throwaway products waste) are critical to the planet's survival. |
| PVS4 | Sustainable waste management practices are critical, in my opinion, to saving the earth. |
| PVS5 | Environmentally responsible consumption, on the whole, provides good value for money. |
| PUS1 | Food waste composts produce similar yields than traditional fertilizers. |
| PUS2 | Food waste composts produce similar yields than organic fertilizer available in the stores. |
| PUS3 | It takes very little time and effort to compost food wastes. |
| PUS4 | Food waste composting is critical to prevent ecological degradation. |
| PUS5 | Food waste composting is beneficial for our health and/or quality of life |
| AOC1 | In my opinion, food waste composting is a major way to reduce pollution |
| AOC2 | I consider that food waste composting creates a better environment for future generations |
| AOC3 | I believe that food waste composting is a major way to reduce wasteful use of landfills |
| AOC4 | I consider that food waste composting is a major way to conserve natural resources |
| AOC5 | I think food waste composting is a major way to prevent ecological degradation. |
| AOR1 | While eating at home, I feel responsible for food waste composting |
| AOR2 | I feel jointly responsible for the negative consequences of food waste. |
| AOR3 | I feel jointly responsible for ecological damage caused by my failure to compost food waste while eating in a restaurant. |
| AOR4 | We are jointly responsible for the environmental deterioration caused by our failure to compost food waste. |
| AOR5 | We are jointly responsible for making environmentally sustainable consumption choices. |
| SUN1 | People whose opinions I value would approve of my engagement in food waste composting. |
| SUN2 | People whose opinions I value approve of my engagement in recycling and composting food waste. |
| SUN3 | Most people who are important to me think I should do whatever I can to prevent food waste dumping. |
| SUN4 | Most people who are important to me would want me to take action to prevent food waste dumping. |
| SUN5 | People whose opinions I value would prefer that I do whatever I can to compost food wastes. |
| ANG1 | I feel guilty that I cannot prevent food waste while eating at home or in a restaurant. |
| ANG2 | I feel guilty about not recycling and/or composting food waste. |
| ANG3 | I feel guilty due to not composting food waste and buying non-organic fertilizers. |
| ANG4 | I feel guilty about throwing food waste away, which contributes to ecological degradation. |
| ANG5 | I feel guilty about the environmental issues that have occurred because of dumping food waste. |
| ATT1 | In my opinion, food waste composting is a great alternative to regular fertilizer. |
| ATT2 | Using food waste compost boosts my chances of producing agricultural products of the expected quality. |
| ATT3 | Increasing agricultural yield through using food waste compost is a good idea. |
| ATT4 | There are various advantages of food waste composting. |
| ATT5 | Food waste composting allows you to save on farming costs. |
| FWI1 | I will try to compost food waste. |
| FWI2 | I intend to not throw food waste away while eating at home or in a restaurant. |
| FWI3 | I will try to compost every time I have any food waste. |
| FWI4 | I intend to change my daily routines by including the time for composting food waste. |
| FWI5 | I intend to contribute to a solution to the problem caused by food waste. |
| FWB1 | I began composting food waste by setting aside some time each week for it. |
| FWB2 | I started composting food waste after learning how to compost food. |
| FWB3 | I started composting food waste as it provide an environmentally friendly alternatives to conventional fertilizers |
| FWB4 | I began composting food waste to save money on conventional fertilizers |
| FWB5 | I started composting food waste to prevent environmental deterioration. |

**Note:** PVS: Perceived value on Sustainability, PUS: Perceived usefulness of food waste compost, AOC: Awareness of Consequences, AOR: Ascription of Responsibility, SUN: Social Influence, ANG: Anticipated Guilt, ATT: Attitude towards food waste composting, FWI: Food waste composting Intention, FWB: Food waste composting Behavior
